# Clinical and serological profile of asymptomatic and non-severe symptomatic COVID-19 cases: Lessons from a longitudinal study in primary care in Latin America

**DOI:** 10.3399/bjgpopen20X101137

**Published:** 2021-01-13

**Authors:** Klaus Puschel, Catterina Ferreccio, Blanca Peñaloza, Katia Abarca, Maria-Paulina Rojas, Alvaro Tellez, Philippa Moore, Ana Maria Cea, Carlos Wilson, Vicente Cid, Joaquin Montero

**Affiliations:** 1 Professor, Department of Family and Community Medicine, School of Medicine, Pontificia Universidad Católica de Chile, Santiago, Chile; 2 Professor, Depatment of Public Health, School of Medicine, Pontificia Universidad Católica de Chile, Santiago, Chile; 3 Associate Professor, Family and Community Medicine, School of Medicine, Pontificia Universidad Católica de Chile, Santiago, Chile; 4 Professor, Department of Infectious Pediatric Diseases, School of Medicine, Pontificia Universidad Católica de Chile, Santiago, Chile; 5 Associate Professor, Department of Family and Community Medicine, School of Medicine Pontificia Universidad Católica de Chile, Santiago, Chile; 6 Professor, Department of Family and Community Medicine, School of Medicine, Pontificia Universidad Católica de Chile, Santiago, Chile; 7 Clinical Nurse, Family and Community Medicine, Pontificia Universidad Catolica de Chile School of Medicine, Santiago, Metropolitana, Chile; 8 Resident, Family and Community Medicine, Pontificia Universidad Catolica de Chile School of Medicine, Santiago, Metropolitana, Chile; 9 Clinical Research Assistant, Department of Public Health, School of Medicine, Pontificia Universidad Católica de Chile, Santiago, Chile; 10 Statistical Research Assistant, Department of Public Health, School of Medicine, Pontificia Universidad Católica de Chile, Santiago, Chile; 11 Professor, Department of Family and Community Medicine, School of Medicine, Pontificia Universidad Católica de Chile, Santiago, Chile

**Keywords:** COVID-19, coronavirus, primary health care, Latin America

## Abstract

**Background:**

Chile has one of the highest incidences of COVID-19 infection in the world. Primary care can play a key role in early detection and containment of the disease. There is a lack of information on the clinical profile of patients with suspected COVID-19 in primary care, and controversy on the effectiveness of rapid serologic tests in the diagnosis and surveillance of the disease.

**Aim:**

To assess the effectiveness of rapid serologic testing in detection and surveillance of COVID-19 cases in primary care.

**Design & setting:**

A longitudinal study was undertaken, which was based on a non-random sample of 522 participants, including 304 symptomatic patients and 218 high-risk asymptomatic individuals. They were receiving care at four primary health clinics in an underserved area in Santiago, Chile.

**Method:**

The participants were systematically assessed and tested for COVID-19 with reverse transcriptase-polymerase chain reaction (RT-PCR) and serology at baseline, and were followed clinically and serologically for 3 weeks.

**Results:**

The prevalence rate of RT-PCR confirmed COVID-19 cases were 3.5 times higher in symptomatic patients (27.5%; 95% confidence interval [CI] = 22.1 to 32.8) compared with asymptomatic participants (7.9%; 95% CI = 4.3 to 11.6). Similarly, the immune response was significantly different between both groups. Sensitivity of serologic testing was 57.8% (95% CI = 44.8 to 70.1) during the third week of follow-up and specificity was 98.4% (95% CI = 95.5 to 99.7).

**Conclusion:**

Rapid serologic testing is ineffective for detecting asymptomatic or non-severe cases of COVID-19 at early stages of the disease, but can be of value for surveillance of immunity response in primary care. The clinical profile and immune response of patients with COVID-19 in primary care differs from those in hospital-based populations.

## How this fits in

Chile had one of the highest incidences of COVID-19 in the world. Primary care can play a key role in early detection of cases and containment of the epidemic. The clinical and serological profile of COVID-19 has not been thoroughly evaluated in the large population of asymptomatic or non-severe symptomatic cases seen in primary care. The immune response of asymptomatic and non-severe cases is significantly lower than that reported in severe cases. Symptoms profile also differs in both populations. Rapid serologic testing is ineffective for detecting asymptomatic or non-severe symptomatic cases of COVID-19, but can be of value for surveillance of immunity response at the primary care and community levels.

## Introduction

The pandemic of COVID-19 emerged strongly in Latin America; by mid-July 2020 the number of new daily cases surpassed that of the US and Europe with Brazil, Peru, and Chile leading the incidence rates. Chile has one of the highest prevalence rates of COVID-19 in the world; higher than Brazil, the US, or the UK as of 31 August 2020.^1^ The stabilisation of incidence rates reported in Chile and in other countries highlights the relevance of finding effective early diagnostic and surveillance strategies at the community level to contain the epidemic, and start a safe unlock process.

Primary health care is at the frontline of the epidemic interface between the community and healthcare services.^2^ Over 80% of COVID-19 cases are non-severe cases that remain in the community and are detected and surveilled in primary care.^3^ The clinical and serological profile of asymptomatic or non-severe cases that present with mild symptoms has not been addressed thoroughly. The great majority of studies evaluating the clinical and serological profile of the COVID-19 infected population have been conducted in hospitalised patients with severe disease.^4–6^ Consistent information on the clinical and serological profile of non-severe COVID-19 cases could contribute to early diagnosis, contact tracing, and effective isolation strategies. As lockdown strategies are reduced, because of a decrease in cases or social exhaustion, the role of primary care services could be essential for effective detection and containment of new outbreaks.

Effective testing and tracing of COVID-19 cases is a key factor in the successful strategies for controlling the epidemic at the primary care level. Accuracy, acceptability, affordability, and results availability of the test at the point of care are essential requirements for effective testing.^7^ The standard RT-PCR test has important limitations as a massive test strategy at the primary care level given that it requires a special sampling procedure, central lab processing, and has a latency of 24–72 hours for the results. Latency time of the test results can be especially problematic in an epidemic scenario, where instant results are needed to implement immediate isolation measures. In addition, sensitivity of RT-PCR declines significantly after 7–8 days of infection, with reported false negative rates of 30%–40%.^8^

Rapid serologic tests have the potential to be an alternative to face the challenge of detection and tracing cases in highly demanded primary care clinics in an epidemic scenario. However, high variability in the accuracy of serologic tests has been reported. In a recent meta-analysis of 38 studies, which included 7848 participants, Kontou *et al* ^9^ found wide sensitivity rates that varied from 56%–83% and specificity rates that ranged from 91%–99%, depending on the characteristics of the serologic test used and the population studied. Many studies included in the meta-analysis did not report clinical severity and were underpowered. Similar results were found in a recent meta-analysis conducted by Lisboa Bastos *et al* ^10^ that analysed 40 studies, although only two of them included outpatients and point-of-care testing. These results do not permit decision makers to draw consistent conclusions on the real value of serologic testing in primary care.

This study assessed the demographic, clinical, and serological profile of non-severe symptomatic and asymptomatic cases of COVID-19 in the primary care setting. The study evaluated the accuracy of rapid serologic testing in the detection and follow-up of COVID-19 infections in primary care.

## Method

### Population and sampling

The study was conducted in four primary healthcare clinics in the south-east area of Santiago, Chile. This area has an estimated population of 1 million people of middle and low socioeconomic status. A non-random sample of 522 individuals was included in the study, 304 of these were symptomatic adults who consulted at these clinics between 8 April and 14 May 2020. They fulfilled the criteria defined by the World Health Organization of suspected cases or had any respiratory symptoms at the time of the visit. In addition, 218 asymptomatic individuals belonging to high-risk groups, such as close contacts of confirmed cases, primary care health personnel, or prison officers working in high-risk environments in the study area, were also invited to participate in the study.

### Procedures

Patients who agreed to participate in the study were assessed clinically using the national clinical guideline recommended for suspected cases.^11^ The guideline included assessing for demographic and risk factors (for example, smoking, high blood pressure, diabetes), chronic diseases (for example, asthma, chronic obstructive pulmonary disease [COPD], cardiovascular disease), medications (for example, angiotensin receptor blockers [ARBs], non-steroidal anti-inflammatory drugs [NSAIDs]) respiratory (for example, sore throat, cough, fever, dyspnoea) and other symptoms (myalgia, headache, anosmia, dysgeusia, gastrointestinal symptoms). It also included a physical exam and general laboratory testing or imaging as required.

At entry (day 1), all participants were tested for COVID-19 through a standard RT-PCR test using a nasopharyngeal swab. The samples were processed at the Microbiology Laboratory, UC-Christus Health Network. Additionally, participants were tested with the rapid serologic test. The test was designed to identify serum immunoglobulin M (IgM) and immunoglobulin G (IgG) from a finger capillary blood that was taken using a point-of-care lateral flow immunoassay (LFIA) COVID-19 test (Acro-Biotech). Results were obtained 10 minutes after the finger stick. Participants were re-evaluated at day 7 if they tested PCR-negative at entry and all were retested and a received a brief clinical assessment after day 14.

### Analysis

A stratified analysis was conducted of symptomatic and asymptomatic participants, and estimated sensitivity, specificity, and likelihood ratios for serologic tests at baseline and follow-up. Associations were explored between risk factors, symptoms, and RT-PCR with serologic results with multivariate logistic regression using R (version 3.5.1) and Stata (version 15).

## Results

The four groups included in the study differed in demographic characteristics such as age, sex, and also in the presence of chronic diseases ( Table 1). Symptomatic patients were older (42.5 years and 33.6 years, *P*<0.001), used more medications (28.1% and 10.5%, *P*<0.001) and had a higher history of chronic respiratory diseases (8.7% and 1.8%, *P*<0.05) compared with all asymptomatic participants. They were similar regarding prevalence of smoking, high blood pressure, and diabetes. The median interval between the first symptom and the clinical assessment in the symptomatic group was 3 days and the most frequent symptoms were headache, dry cough, and sore throat. The positivity of RT-PCR test was 3.5 times higher in symptomatic versus asymptomatic groups (27.5% [95% CI = 22.1 to 32.8] versus 7.9% [95% CI = 4.3 to 11.6]; *P*<0.001). Similarly, positivity of IgM or IgG during the study period among symptomatic patients was 3.8 times higher than the asymptomatic participants (18.5% [95% CI = 14.1 to 23.0] versus 4.9 [95% CI = 1.7 to 8.1]; *P*<0.001).

**Table 1.** Characteristics of study participants (*n* = 522)

	Symptomatic patients(*n* = 304, 58.2%)	Asymptomatic patients (*n* = 218, 41.8%)
	Prison officers(*n* = 139, 26.6%)	Health workers(*n* = 39, 7.5%)	Contacts(*n* = 40, 7.7%)
Charateristics	Mean ± SD / Prevalence % (95% CI)
Mean age, years, mean ± SD	42.5 ± 15.3	32.7 ± 6.8	33.0 ± 9.0	37.3 ± 13.8
Women (*n* = 217)	53.6 (48.0 to 59.2)	5.8 (1.9to 9.6)	76.9 (63.7 to 90.1)	40.0 (24.8 to 55.2)
Current smoker (*n* = 149)	34.8 (28.9 to 40.8)	37.3 (27.5to 47.2)	22.6 (6.7 to 38.5)	27.5 (12.3 to 42.7)
**Medications**				
ARBs (*n* = 37)	9.0 (6.1 to 11.9)	–	10.1 (0.0 to 21.6)	17.0 (5.3 to 28.7)
ACE inhibitors (*n* = 14)	4.0 (1.9 to 6.1)	–	6.1 (0.0 to 17.0)	–
NSAIDs (*n* = 16)	3.1 (1.6 to 4.5)	–	–	–
Oral steroids (*n* = 5)	1.3 (0.0 to 2.6)	–	–	2.6 (0.0 to 7.7)
Inhalator steroids (*n* = 16)	4.8 (2.5to 7.0)	–	–	3.7 (0.0 to 10.6)
**Chronic diseases**				
High blood pressure (*n* = 76)	15.4 (12.1 to 18.7)	9.8 (28.8to 16.8)	17.8 (5.5 to 30.1)	13.7 (4.1 to 23.2)
Diabetes (*n* = 31)	8.4 (5.6 to 11.1)	–	5.9 (0.0 to 16.0)	7.1 (0.0 to 15.8)
Asthma or COPD (*n* = 33)	8.7 (5.6 to 11.8)	1.0 (0.0 to 3.1)	–	5.3 (0.0 to 12.4)
Cardiovascular disease (*n* = 9)	1.7 (0.7 to 2.7)	–	–	–
Cancer (*n* = 6)	1.1 (0.2 to 2.1)	–	–	–
Prevalence RT-PCR positive (*n* = 100)	27.5 (22.1 to 32.8)	7.9 (3.4 to 12.3)	8.8 (0.0 to 18.4)	7.5 (0.0 to 15.6)
Prevalence of IgM(+) or IgG (+) ever (*n* = 70)	18.5 (14.1 to 23.0)	4.4 (0.5 to 8.2)	3.2 (0.0 to 9.4)	7.8 (0.0 to 16.2)
IgM (+) or IgG (+) by days of initial symptoms^a^:				
Baseline (median: 3 days; [0–21]) (*n* = 25)	5.7 (3.1 to 8.3)	3.3 (0.0 to 6.8)	3.9 (0.0 to 11.3)	2.7 (0.0 to 7.7)
Follow-up 1 (median: 11 days; [6–24]) (*n* = 7)^b^	1.9 (0.0 to 3.9)	1.0 (0.0 to 3.1)	2.5 (0.0 to 7.7)	3.0 (0.0 to 8.9)
Follow-up 2 (median: 19 days [12–33]) (*n* = 48)^c^	18.0 (13.3 to 22.6)	–	–	5.6 (0.0 to 13.2)
**Initial symptoms profile (only symtpomatic patients)**				
**Symptoms**	**Prevalence % (** **95%** **CI** **)**	**Symptoms**	**Prevalence % (** **95%** **CI** **)**	
Cephalea (*n* = 202)	66.9 (61.7 to 72.1)	Fatigue (*n* = 49)	16.2 (12.1 to 20.4)	
Dry cough (*n* = 141)	46.7 (41.1 to 52.3)	Productive cough (*n* = 44)	14.6 (10.7 to 18.5)	
Sore throat (*n* = 137)	45.4 (39.8 to 50.9)	Fever (*n* = 44)	14.6 (10.6 to 18.5)	
Myalgia (*n* = 128)	42.4 (36.8 to 47.9)	Nasal congestion (*n* = 42)	13.9 (10.0 to 17.8)	
Feverish sensation (*n* = 83)	27.5 (22.5 to 32.5)	Chest pain (*n* = 40)	13.2 (9.4 to 17.0)	
Diarrhoea (*n* = 57)	18.9 (14.5 to 23.2)	Anosmia (*n* = 30)	9.9 (6.6 to 13.3)	
Disnea (*n* = 53)	17.5 (13.4 to 21.7)	Dysgeusia (*n* = 22)	7.3 (4.4 to 10.2)	

^a^Prevalence data are adjusted by age, and sex using logistic regression. Age mean data are adjusted by sex using linear regression. ^b^Follow-up 1: follow-up in first time period *n* = 381. ^b^follow-up 2: follow-up in second time period *n* = 447. ARBs = angiotensin receptor blockers; ACE = angiotensin-converting-enzyme; NSAIDs = non-steroidal anti-inflammatory drugs; COPD = chronic obstructive pulmonary disease; RT-PCR = reverse transcriptase-polymerase chain reaction; IgM = immunoglobulin M; IgG = immunoglobulin G

**Figure 1.**
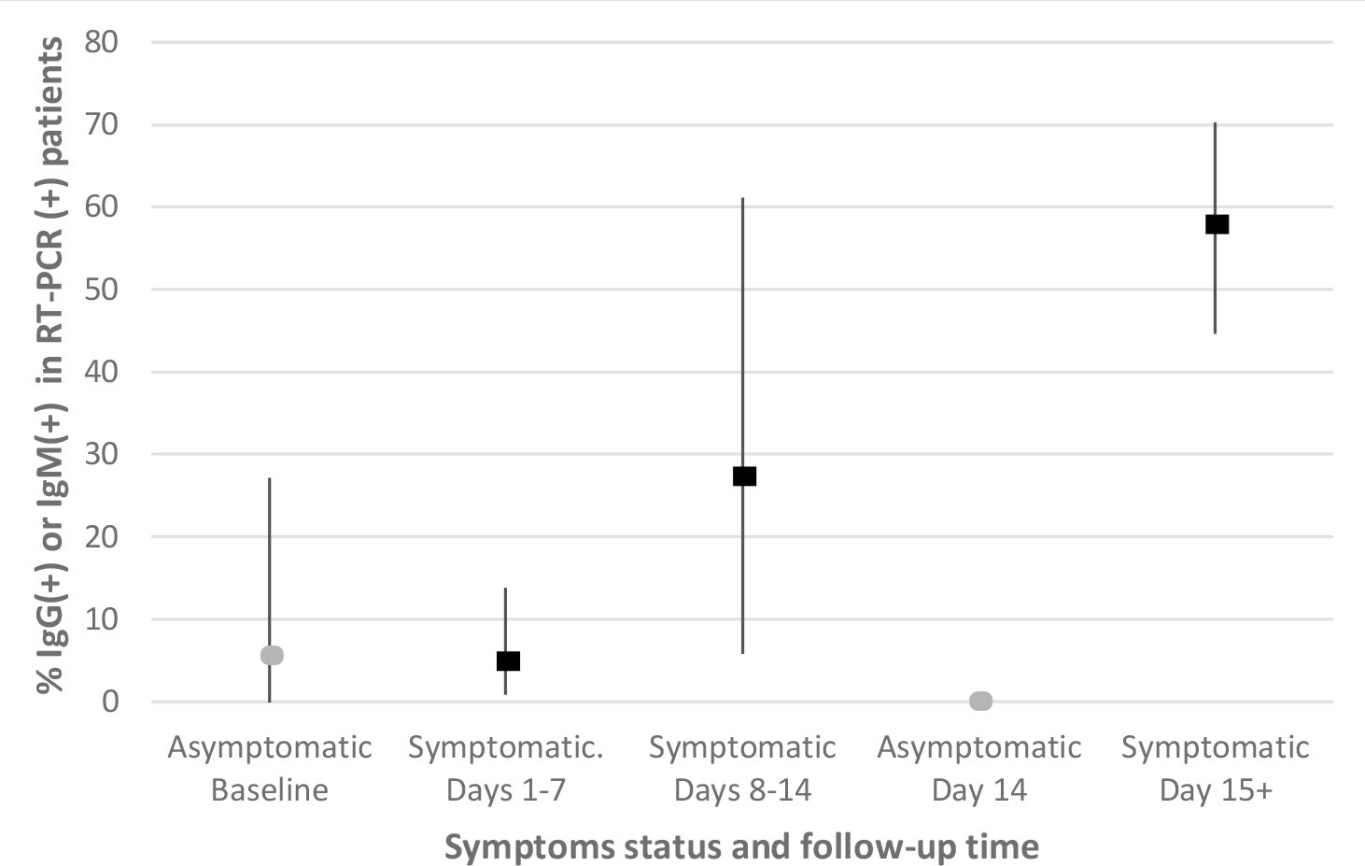
Positive rate (%) of IgM or IgG in symptomatic and asymptomatic patients with positive RT-PCR over time. RT-PCR = reverse transcriptase-polymerase chain reaction

The relative sensitivity, specificity, and likelihood ratios of the serologic tests compared with RT-PCR in symptomatic patients over time are shown in Table 2. The relative sensitivity of serology increased from 4.9% in the first week to 57.8% at day 14. The specificity of serological tests remained over 96% throughout the study. The positive likelihood ratio was 36.6 after 14 days of initial symptoms. The sensitivity of the serologic tests in asymptomatic participants remained very low, below 5% during the follow-up period and specificity remained over 97%. Figure 1 presents the positivity rate (PR) of serologic tests in symptomatic and asymptomatic patients with positive RT-PCR results over time. Main predictors of a positive serology among symptomatic patients were: older age (PR = 1.03, 95% CI = 1.02 to 1.05) , female sex (PR = 1.63, 95% CI = 1.06 to 2.51), and dry cough (PR = 1.79, 95% CI = 1.14 to 2.81).

**Table 2.** Relative sensitivity, specificity, and likelihood ratios associated with serologic tests compared with RT-PCR test results in symptomatic patients by time period

**Symptomatic patients,** **≤** **7** **days** **since first symptom**					
	RT-PCR (+)	RT-PCR (-)	Total	Sensitivity	4.9% (1.0% to 13.7%)
IgM or IgG (+)	3	5	8	Specificity	96.9% (92.9% to 99.0%)
IgM or IgG r (-)	58	157	215	LR+	1.59 (0.39 to 6.47)
Total	61	162	223	LR-	0.98 (0.92 to 1.05)
**Symptomatic patients,** **8** **–** **14** **days**					
	RT-PCR (+)	RT-PCR (-)	Total	Sensitivity	27.3% (6.0% to 61.0%)
IgM or IgG (+)	3	2	5	Specificity	98.7% (95.4% to 99.8%)
IgM or IgG r (-)	8	154	162	LR+	21.27 (3.96 to 114.3)
Total	11	156	167	LR-	0.74 (0.51 to 1.06)
**Symptomatic** **patients** **≥** **15** **days**					
	RT-PCR (+)	RT-PCR (-)	Total	Sensitivity	57.8% (44.8% to 70.1%)
IgM or IgG (+)	37	3	40	Specificity	98.4% (95.5% to 99.7%)
IgM or IgG r (-)	27	187	214	LR+	36.6 (11.69 to 114.71)
Total	64	190	254	LR-	0.43 (0.32 to 0.57)

RT-PCR = reverse transcriptase polymerase chain reaction; LR = likelihood ratio; IgM = immunoglobulin M; IgG = immunoglobulin G

## Discussion

### Summary

This study shows that LFIA serologic testing is not effective for early diagnosis of COVID-19 in primary health care given its low accuracy in detecting cases. The immune response of asymptomatic and non-severe cases is significantly lower than that reported in severe cases. The symptom profile also differs in both populations. LFIA serologic testing might be of value for surveillance in primary health care and in the community given its high specificity and increasing detection rate after 2 weeks of infection.

### Strengths and limitations

This study targets a key population for controlling the epidemic that has not been addressed thoroughly. Participants were non-randomly selected and, therefore, do not represent an homogenous population. They were selected on clinical criteria of symptoms suggestive of COVID-19 disease or on epidemiological criteria of high-risk populations. The criteria used for including participants were similar to that recommended for COVID-19 testing in primary health care and in the community,^12^ and the study reflects the reality of this level of care. The LFIA tests used in this study had the least sensitivity of the serologic tests available for rapid testing. The test was chosen because it was the most widely used at the time, was affordable, rapidly available, and the reported accuracy was acceptable. These are essential characteristics to value the effectiveness of a diagnostic test in primary care.^7^

### Comparison with existing literature

The sero-prevalence results of the study are similar to those observed in a community-based study conducted in England, which reported 7% of sero-prevalence in May 2020,^13^ and those reported in the surveillance programme in England, which found a range from 4% (South East) to 16% (London) from week 12 to week 20 in a mixed adult population.^14^ In contrast to severe COVID-19 cases where fever is the predominant symptom present in over 80% of cases,^4^ in the non-severe population fever was present in only 15% of cases. Dry cough was the predominant symptom in the symptomatic population with a significant predictive value for infection.

Accuracy assessment of serologic testing in the study revealed a low relative sensitivity that ranged from 8% during the first week of symptoms or contact date to 58% during the third week of follow-up. Specificity remained over 95% along the study when compared with RT-PCR results. The low sensitivity observed in this study contrasts with most reports that showed sensitivity rates over 80%,^9^ and also with the manufactured company data of the serologic point-of-care test used in this study that reported relative sensitivity values of 100% for IgM and 85% for IgG.^15^ These results highlight the difference in the intensity of humoral response in non-severe cases versus in the severe cases that have also been reported in other studies.^16,17^

The results of this investigation are consistent with evidence showing that there is a significant seroconversion rate of IgG after 14 days of symptom onset, but that antibody levels in non-severe cases are low or maybe undetectable.^16,18,19^ The increase in the seroconversion rate over time observed was associated, as expected, with a raise in the sensitivity of the test after the second week, which has also been observed in many other studies.^10^The available evidence shows that viral dynamics and serologic response are very different when comparing asymptomatic, non-severe, or severe COVID-19 cases. Liu *et al* ^20^ found that the mean viral load of severe cases was around 60 times higher than that detected in mild cases in a group of 76 patients admitted to the Nanchang University Hospital in China. Similarly, Long *et al* ^5^ found that severely ill patients had significantly higher IgG levels compared with non-severe patients in a cohort of 250 hospitalised patients in Hubei, China. The results are also consistent with evidence showing a very low seroconversion in asymptomatic versus symptomatic patients and high percentage of seronegative conversion after the early convalescent phase.^16,17^ The present study shows a relatively low detectable seroconversion rate in about 60% of the non-severe population after the second week of infection with a significant difference between symptomatic and asymptomatic high-risk patients. By providing specific information in a population of non-severe and asymptomatic cases, this study contributes to the growing evidence of a gradient between severity of the disease, viral load, and immune response.

The characteristics of the test used for antibody detection is another important source of variability in the detection rate of COVID-19 infection. In a meta-analysis of 37 studies conducted by Kountou *et al*,^9^ specificity of the four techniques analysed were over 90%. The results are consistent with these estimates. However, the sensitivity of the tests were much more variable and LFIAs were the least sensitive with a range of 75%–80%. The National COVID Advisory Panel at the UK found that sensitivity estimates for LFIAs ranged from 55%–70% versus 65%–85% for enzyme-linked immunosorbent assays (ELISAs) when compared with RT-PCR.^21^ However, most of the studies analysed by both groups included only hospitalised and severe patients. The present study found a sensitivity of 57.8% at the third week of infection in the population of non-severe symptomatic and asymptomatic cases.

### Implications for practice

Early detection of non-severe and asymptomatic COVID-19 cases has been defined as a key strategy to control the epidemic,^14^ given that these cases account for about 80% of the infected population.^3^ The present study targets this population and shows that given its low sensitivity, LFIA tests are not effective to detect COVID-19 in early stages in non-severe symptomatic or high-risk asymptomatic populations. As such, LFIAs appear to be of little use in the early diagnosis of COVID-19 at a primary care level. Other techniques, such as ELISA, or the RT-PCR saliva test, might be more effective techniques for early diagnosis, but at a higher cost.

In spite of the limitations of the serologic testing observed in this study for early diagnosis in a population with non-severe COVID-19 infection, LFIA could be of value for surveillance at a community level given the high specificity observed after the second week of infection. Applying the rapid test in a closed community, such as a school, workplace, or neighbourhood, could help to estimate the prevalence of past disease, magnitude of recent outbreak, and the real need for new testing. The presence of detectable levels of antibodies means that infection has already occurred with a very high probability, even in individuals with low intensity of symptoms. It can provide an estimate of the immune status of a local community.^22^ The experience in Singapore^23^ and in Taiwan^24^ showed that serologic testing is a complementary strategy for surveillance given that RT-PCR fails to detect cases beyond the first week of infection. Serologic testing can provide valuable information on the attack and transmission rates at the community level.^22^ These are key factors to consider for improving containment of an outbreak or defining unlocking measures at a local level.^13^ The present study shows an increasing serologic trend beyond the second week of infection, and very low false positive rates in symptomatic and asymptomatic cases. These results can contribute to improving surveillance of the disease at the community level.
